# Supplementary material for: DMRforPairs: identifying Differentially Methylated Regions between unique samples using array based methylation profiles
Source: BMC Bioinformatics. 2014 May 15;15:141. doi: 10.1186/1471-2105-15-141 (PMC4046028; doi:10.1186/1471-2105-15-141)
Supplement: Additional file 2 — DMRforPairs output for the comparison of A431-MCF7 and NA17018-NA17105. Please start from the HTML files in each folder. Available via the BMC Bioinformatics website. [file 1471-2105-15-141-S2.zip › 1394847754114233_MOESM2_ESM/A431_MCF7/figures/10436.pdf]

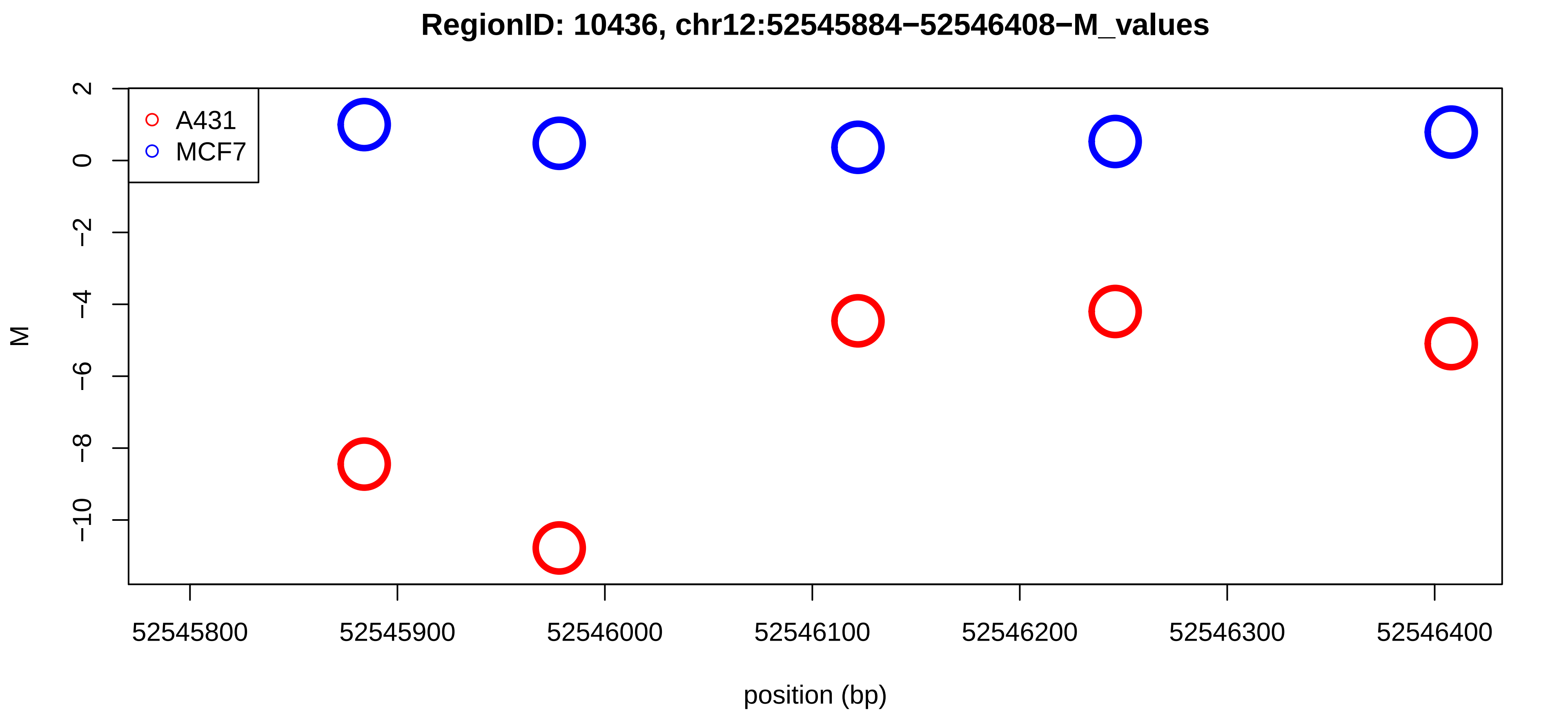

RegionID: 10436, chr12:52545884−52546408−M_values
A431
MCF7
M
2
0
−2
−4
−6
−8
−10
52545800
52545900
52546000
52546100
52546200
52546300
52546400
position (bp)

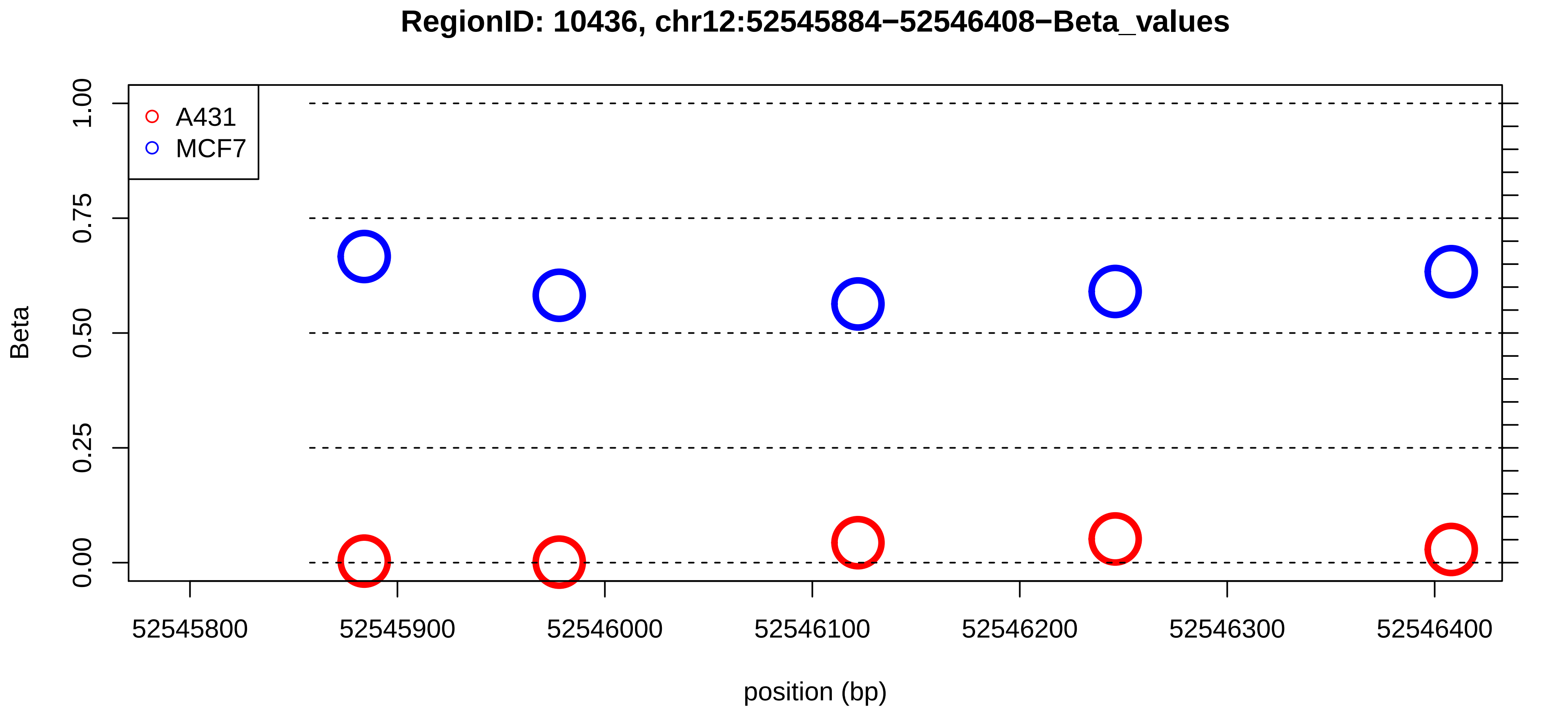

RegionID: 10436, chr12:52545884−52546408−Beta_values
A431
MCF7
Beta
1.00
0.75
0.50
0.25
0.00
52545800
52545900
52546000
52546100
52546200
52546300
52546400
position (bp)

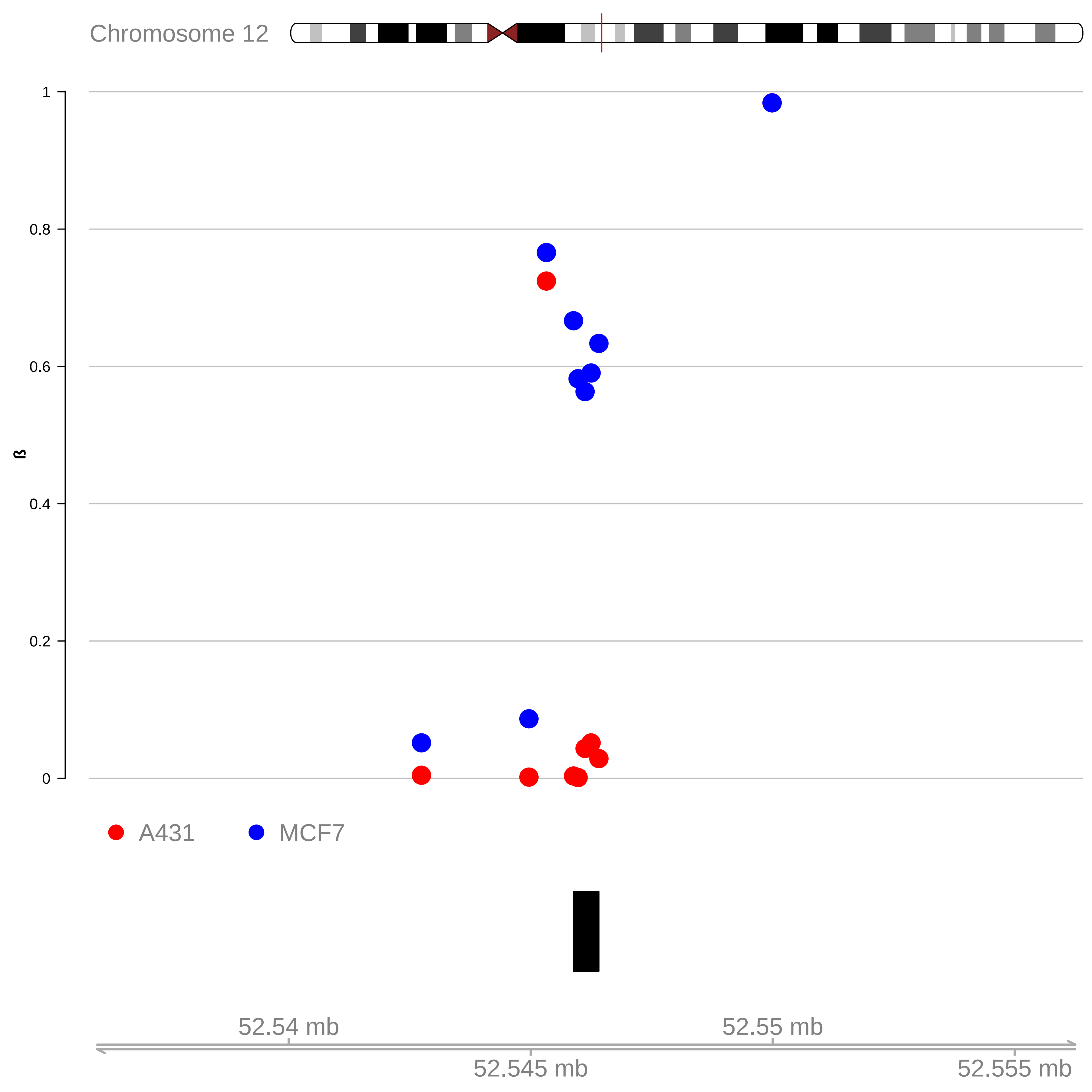
Chromosome 12
1
0.8
0.6
ß
0.4
0.2
0
A431
MCF7
52.54 mb
52.55 mb
52.545 mb
52.555 mb
